# Study on the priority of coronary arteriography or therapeutic hypothermia after return of spontaneous circulation in patients with out-of-hospital cardiac arrest: results from the SOS-KANTO 2012 study

**DOI:** 10.1007/s11739-015-1378-2

**Published:** 2016-02-01

**Authors:** Shuichi Hagiwara, SOS-KANTO 2012 study group

**Affiliations:** Department of Emergency Medicine, Gunma University Graduate School of Medicine, 3-39-22 Showa-machi, Maebashi, Gunma 371-8511 Japan

**Keywords:** Therapeutic hypothermia, Coronary angiography, Out-of-hospital cardiac arrest

## Abstract

Many emergency physicians struggle with the clinical question of whether to perform therapeutic hypothermia (TH) or coronary angiography (CAG) first after return of spontaneous circulation (ROSC) in patients with out-of-hospital cardiac arrest (OHCA). We analyzed the results of the SOS-KANTO 2012 study, which is a prospective, multicenter (67 emergency hospitals), observational study about OHCA conducted between January 2012 and March 2013 (*n* = 16,452). We compared two groups: the group in which TH was first performed (TH group), and the group in which CAG was performed first (CAG group) within 24 h after arrival. Two hundred and twenty-one patients were treated TH and CAG (TH group, 76 patients; CAG group, 145 patients). In addition, we selected patients who underwent coronary treatment. 164 patients underwent coronary treatment after CAG (TH group, 52 patients; CAG group, 112 patients). In patients in whom TH and CAG and coronary artery treatment were done, 42 patients (55.3 %) in the TH group and 86 patients (59.3 %) in the CAG group survived at 90 days. The cerebral performance category (CPC) 1 and 2 were 26.3 % (20 patients) in TH group, and 31.0 % (45 patients) in CAG group. In patients in whom TH and CAG with coronary artery treatment were performed, 29 patients (55.8 %) in the TH group and 64 patients (57.1 %) in the CAG group survived at 90 days. The rates of CPC 1 and 2 were 26.9 % (14 patients) in TH group, and 23.2 % (26 patients) in CAG group. There was no significant difference in 90-day survival between the two groups although it tended to be better in the CAG group than in the TH group. Whether TH or CAG was performed first did not affect the 90-day survival and 30-day neurological situation among patients with ROSC after OHCA.

## Introduction

Out-of-hospital cardiac arrest (OHCA) is one of the greatest problems of community and public health. For patients with OHCA to have a good prognosis, it is essential not only to resuscitate precisely, but also to perform post-cardiac arrest care appropriately after return of spontaneous circulation (ROSC).

In post-cardiac arrest care, therapeutic hypothermia (TH) is a helpful approach for protection of the brain and other organs in patients who remain comatose after ROSC [1, 2]. TH may be considered for comatose adult patients with ROSC after OHCA according to the 2010 American Heart Association (AHA) Guidelines for Cardiopulmonary Resuscitation (CPR) and Emergency Cardiovascular Care (ECC) [3]. Those guidelines recommend that the body temperature should be cooled to 32–34 °C for 12–24 h in TH.

On the other hand, the usefulness of emergent coronary angiography (CAG) in survivors of OHCA has already been reported [4, 5], and the 2010 AHA Guidelines for CPR and ECC insist that emergent CAG may be reasonable because of the high incidence of acute coronary ischemia in OHCA patients [3]. In particular, percutaneous coronary intervention (PCI), alone or as part of a bundle of care, is associated with improved myocardial function [4] and neurological outcomes [5, 6]. However, the priority of TH or CAG has not been established yet. So there are doctors who are worried about the risk of pulmonary edema in using low temperature hypothermia [7], and do not believe there is convincing evidence to support the use of hypothermia prior to CAG. Such doctors think TH (including cooling blanket and ice packs) should start after CAG/PCI. This study was projected for such doctors to provide more evidence of early or simultaneous TH and CAG.

A Survey of Survivors of OHCA in the Kanto district of Japan 2012 study (SOS-KANTO 2012 study) was performed by the Japanese Association for Acute Medicine in Kanto district between January 2012 and March 2013, and included 67 emergency hospitals and emergency medical services units (26 academic medical centers). The SOS-KANTO 2012 study was a prospective, multicenter, observational study about OHCA.

We evaluate the priority of TH or CAG after ROSC in patients with OHCA using the data of the SOS-KANTO 2012 study.

## Methods

The SOS-KANTO 2012 study was a prospective, multicenter (67 emergency hospitals), observational study conducted between January 2012 and March 2013 on patients with OHCA. The protocol of the SOS-KANTO 2012 study was approved without the need for informed consent by the research ethics board of Yokohama City University Medical Center (Yokohama, Kanagawa, Japan; D1402005). The total number of OHCA patients who were transferred to the 67 emergency hospitals (26 academic medical centers), and the emergency hospitals that participated in the SOS-KANTO 2012 study are listed in *Acknowledgments*. The results of SOS-KANTO 2012 study are published in several journals [8–11].

**Fig. 1:**
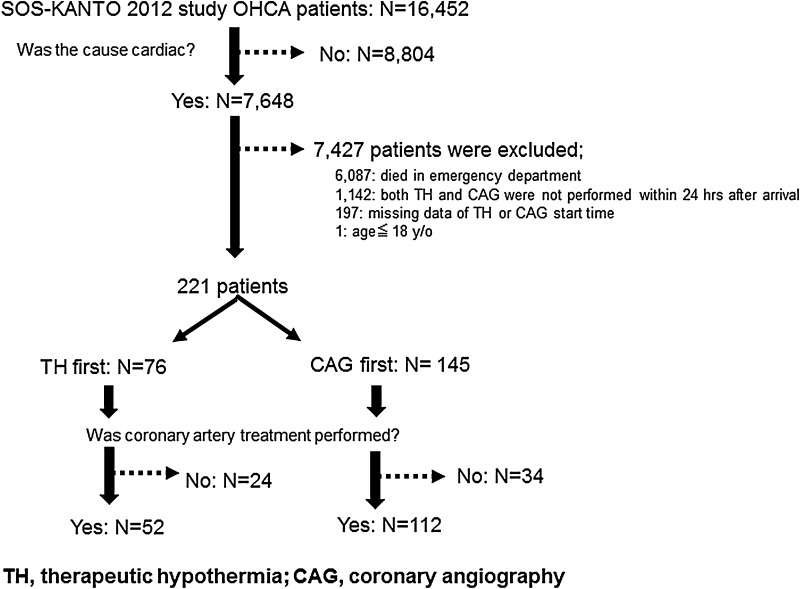
The study flow diagram

The study flow diagram is shown in Fig. 1. First, we selected OHCA patients who underwent both TH and CAG after ROSC within 24 h after arrival at the hospital from all OHCA patients. Patients ≤ 18 years were excluded. Patients with missing data were also excluded. Then, these patients were divided into the following two groups: (1) TH was performed first prior to CAG (TH group), and (2) CAG was performed first prior to TH (CAG group). Next, we analyzed the patients who were treated with coronary artery stenting. In this study, we defined CAG as only coronary artery imaging using catheter.

The indications for CAG and coronary artery treatment in patients with OHCA were decided by emergency physicians and cardiologists at each hospital. TH was defined as maintenance of core body temperature at 32–34 °C for 12–24 h after ROSC, and the methodology of TH was entrusted to each hospital.

The primary outcomes were 90-day survival and neurological status, and the secondary outcomes were 30-day neurological status and complications associated with TH. Neurological status was described by cerebral performance category (CPC) [12, 13]. Good neurological outcome was defined as CPC1 and CPC2.

### Statistical analysis

Differences between the groups were analyzed with Student’s *t* test for normally distributed variables or Mann–Whitney *U* test for non-normally distributed variables, and the Fisher’s exact test for categorical variables. Survival curves were drawn by the Kaplan–Meier method, and were compared with the log-rank test.

IBM SPSS Statistics 22 and EZR (Saitama Medical Center, Jichi Medical University, Saitama, Japan) were used for statistical analyses. EZR is a graphical user interface for R (The R Foundation for Statistical Computing, Vienna, Austria). More precisely, it is a modified version of R commander designed to add statistical functions frequently used in biostatistics [14]. Statistical significance was assumed to be present at a *p* value of <0.05.

## Results

During the study period, 16,452 patients were collected in the SOS-KANTO 2012 study.

As shown in Fig. 1, both TH and CAG were performed within 24 h after arrival at the hospital in 221 patients. TH was performed first in 76 patients (TH group), and CAG was performed first in 145 patients (CAG group). Among them, there were 164 patients in whom the coronary arteries were treated (52 patients in TH group, and 112 patients in CAG group).

Table 1 shows the characteristics of patients in the TH (*n* = 76) and CAG (*n* = 145) groups who are with or without coronary treatment. There were 61 males in TH group, and 123 males in CAG group, respectively (*p* = 1). The mean age ± standard deviation (SD) (years old) of TH group is 60.4 ± 13.6, that of CAG group is 63.1 ± 11.2, respectively (*p* = 0.11). The median time from arrival to CAG start [±, interquartile range (IQR) (min)] in TH group is 88.5 ± 70.5, in CAG group is 70.0 ± 46.0, respectively (*p* = 0.014). The median time from arrival to reach the target body temperature [± IQR (min)] in TH group is 155 ± 249.5, in CAG group is 300 ± 225, respectively (*p* < 0.001). The median time from TH start to reach the target body temperature [± IQR (min)] in TH group is 105 ± 210, in CAG group is 161.5 ± 154.6, respectively (*p* = 0.191). There were 61(80.3 %) patients with witnessed arrest in the TH group, and 115 (79.3 %) patients with witnessed arrest in the CAG group, respectively (*p* = 1). There were 45 (59.2 %) patients with by-stander CPR in the TH group, and 73 (50.3 %) patients with by-stander CPR in the CAG group, respectively (*p* = 0.552). There were 52 (68.4 %) patients who were in ventricular fibrillation/pulseless ventricular tachycardia (VF/VT) in the TH group, and there were 84 (57.9 %) patients that were in VF/VT in the CAG group in the electrocardiogram (ECG) wave form at first, respectively (*p* = 0.496). There were 17 (22.4 %) patients in VF/VT in the ECG wave form at arrival in TH group, and there were 26 (17.9 %) patients in CAG group, respectively (*p* = 0.601). There were 34 (44.7 %) patients who were treated with extra corporeal membrane oxygenation (ECMO) in the TH group, and there were 45 (31.0 %) patients who were treated ECMO in CAG group, respectively (*p* = 0.177). There were 41 (53.9 %) patients who were treated with intra-aortic balloon pumping (IABP) in the TH group, and there were 73 (50.3 %) patients who were treated with IABP in the CAG group, respectively (*p* = 0.809). There were 4 (5.3 %) patients who were treated with blood purification (BP) including hemodialysis (HD) or continuous hemodiafiltration (CHDF) in the TH group, and there were 20 (13.8 %) patients who were treated with BP in the CAG group, respectively (*p* = 0.108).

**Table 1:** Patients’ characteristics of the TH group and CAG group **(**TH and CAG with or without coronary artery treatment was performed in all patients)

	TH group (*N* = 76)	CAG group (*N* = 145)	*p*
Sex (M/F) (*N*)	61/12	123/22	1
Mean age (years old) ± SD	60.4 ± 13.6	63.1 ± 11.2	0.11
Median time from amvai to CAG start [± IQR (min)]	88.5 ± 70.5	70 ± 46	0.014
Median time from amvai to reach the target body temperature [± IQR (min)]	155 ± 249.5	300 ± 225	<0.001
Median time from TH start to reach the target body temperature [± IQR (min)]	105 ± 210	161.5 ± 154.6	0.191
Witnessed [*N* (%)]	61 (80.3)	115 (79.3)	1
by-stander CPR [*N* (%)]	45 (59.2)	73 (50.3)	0.552
ECG waveform at first [*N* (%)]			
VF/VT	52 (68.4)/0 (0)	84 (57.9)/0 (0)	0.496
PEA	7 (9 2)	29 (20 0)	0.085
Asystole	6 (7 9)	8 (5 5)	0.568
Other	10 (13 2)	24 (16 6)	0.698
ECG waveform arrival [*N* (%)]			
VF/VT	17 (22 4)/0 (0)	26 (17.9)/0 (0)	0.601
PEA	14 (18 4)	32 (22 1)	0.732
Asystole	10 (132)	22 (15 2)	0.843
Other	35 (46 1)	65 (44 8)	1
With ECMO [*N* (%)]	34 (44 7)	45 (31 0)	0.177
With IABP [*N* (%)]	41 (53 9)	73 (50 3)	0.809
With BP [*N* (%)]	4 (5 3)	20 (13 8)	0.108

*TH* therapeutic hypothermia, *CAG* coronary angiography, *SD* standard deviation, *IQR*_*t*_ interquartile range, *CPR* cardiopulmonary resuscitation, *ECG* electrocardiogram, *VF*/*VT* ventricular fibrillation pulseless ventricular tachycardia, *PEA* pulseless electrical activity, *ECMO* extracorporeal membrane oxygenation, *IAPB* intra-aortic balloon pumping, *BP* blood purification

Table 2 shows characteristics of the patients who received coronary artery treatment (52 patients in TH group, and 112 patients in CAG group). Fifty patients were treated with catheter, and 2 patients were treated other methods in the TH group, 109 patients were treated with catheter and 3 patients with other methods in the CAG group. There were 44 males in the TH group, and 96 males in the CAG group, respectively (*p* = 1). The mean age ± SD (years old) of the TH group was 62.2 ± 11.3, that of the CAG group was 63.1 ± 11.5, respectively (*p* = 0.652). The median time from arrival to CAG start [± (IQR) (min)] in the TH group was 83.5 ± 65.3, in the CAG group was 69.5 ± 44.8, respectively (*p* = 0.062). The median time from arrival to reach the target body temperature [± IQR (min)] in the TH group is 147 ± 243.8, in the CAG group is 300 ± 232.3, respectively (*p* < 0.001). The median time from TH start to reach the target body temperature [± IQR (min)] in the TH group is 120 ± 192, is 180 ± 150 in the CAG group, respectively (*p* = 0.259). There were 42 (80.8 %) patients with witnessed in the TH group, and 87 (77.7 %) patients with witnessed in the CAG group, respectively (*p* = 0.9). There were 30 (57.7 %) patients with by-stander CPR in the TH group, and 56 (50.0 %) patients with by-stander CPR in the CAG group, respectively (*p* = 0.671). There were 35 (67.3 %) patients who were VF/VT in the TH group, and 62 (55.4 %) in VF/VT in the CAG group in the ECG wave form at first, respectively (*p* = 0.498). There were 10 (19.2 %) patients in VF/VT in the ECG wave form at arrival in the TH group, and there were 20 (17.9 %) patients in the CAG group, respectively (*p* = 0.835). There were 22 (42.3 %) patients who were treated ECMO in the TH group, and there were 38 (33.9 %) patients who were treated with ECMO in the CAG group, respectively (*p* = 0.523). There were 31 (59.6 %) patients who were treated with IABP in the TH group, and there were 64 (57.1 %) patients who were treated with IABP in the CAG group, respectively (*p* = 0.891). There were 3 (5.8 %) patients who were treated with BP in the TH group, and there were 17 (15.2 %) patients who were treated with BP in the CAG group, respectively (*p* = 0.194).

**Table 2:** Patients’ characteristics of the TH group and CAG group (TH and CAG with coronary artery treatment were performed in all patients)

	TH group (*N* = 52)	CAG group (*N* = 112)	*p*
Sex(W/F) (*N*)	44/8	96/16	1
Mean age (years old) ± SD	62.2 ± 1.13	63.1 ± 11.5	0.652
Median time from arrival to CAG start [± IQR (min)]	83.5 ± 65.3	69.5 ± 44.75	0.062
Median time from arrival to reach the target body temperature [± IQR (min)]	147 ± 243 8	300 ± 232.3	<0.001
Median time from TH start to teach the target body temperature [± IQR (min)]	120 ± 192	180 ± 150	0.259
Median time from arrival to coronary reperfusion [± IQR (min)]	162 ± 73*	120 ± 69**	<0.001
Witnessed [*N* (%)]	42 (80.8)	87 (77.7)	0.9
By-stander CPR [*N* (%)]	30 (57.7)	56 (50)	0.671
ECG waveform at first [*N* (%)]			
VF/VT	35 (67.3)/0 (0)	62 (55 4)/0 (0)	0.498
PEA	6 (115)	24 (214)	0.278
Asystole	2 (3.8)	6 (5.4)	1
Other	9 (17.3)	20 (17.9)	1
ECG waveform at arrival [*N* (%)]			
VF/VT	10 (19.2)/0 (0)	20(17.9)/0	0.35
PEA	12 (23.1)	26 (23.2)	1
Asystole	7 (13.5)	16 (14.3)	1
Other	23 (44.2)	50 (44.6)	1
With ECMO [*N* (%)]	22 (42.3)	38 (33.9)	0.523
With IABP [*N* (%)]	31 (59.6)	64 (57.1)	0.891
With BP [*N* (%)]	3 (58)	17 (15.2)	0.194

*TH* therapeutic hypothermia, *CAG* coronary angiography, *SD* standard deviation, *IQR*_*t*_ interquartile range, *CPR* cardiopulmonary resuscitation, *ECG* electrocardiogram, *VF*/*VT* ventricular fibrillation/pulseless ventricular tachycardia, *PEA* pulseless electrical activity, *ECMO* extracorporeal membrane oxygenation, *IAPB* intra-aortic balloon pumping, *BP* blood purification

*N = 37; **N = 87

We could analyze the time from arrival to coronary reperfusion [coronary reperfusion was defined as thrombolysis in myocardial infarction (TIMI) grade 2 or 3] in only 124 patients (TH group: *N* = 37, CAG group: *N* = 87). The median time from arrival to coronary reperfusion [± IQR (min)] is 162 ± 73 in the TH group, and 120 ± 69 in the CAG group, respectively (*p* < 0.001).

### Primary outcomes

**Fig. 2:**
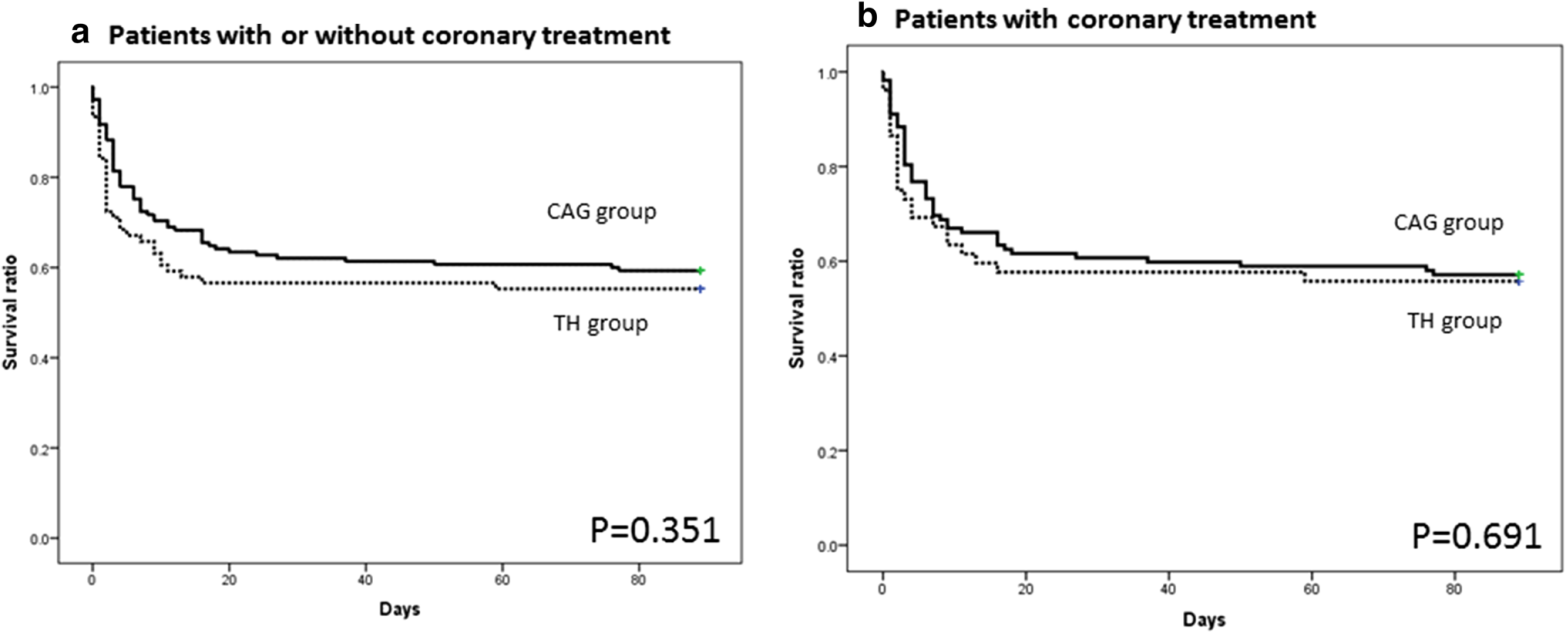
**a** Kaplan–Meier curves of the TH group and CAG group with and without coronary artery treatment. There was no significant difference between the two groups (log-rank test). **b** Kaplan–Meier curves of the TH group and CAG group with coronary artery treatment. There is no significant difference between the two groups (log-rank test). *TH* Therapeutic hypothermia, *CAG* coronary angiography

Figure 2a shows the Kaplan–Meier survival curves of patients in whom TH and CAG with or without coronary artery treatment were done. At 90 days, 42 patients (55.3 %) in the the TH group and 86 patients (59.3 %) in the the CAG group survived. Estimated mean survival day is 52.6 (standard error: 5.8, 95 % confidence interval 41.2–63.9) days in the TH group and estimated mean survival day was 55.3 (standard error: 3.8, 95 % confidence interval 47.8–62.8) days in the CAG group, respectively. Figure 2b shows the Kaplan–Meier survival curves of patients in whom TH and CAG with coronary artery treatment were performed. At 90 days, 29 patients (55.8 %) in the TH group and 64 patients (57.1 %) in the CAG group survive. Estimated mean survival day is 51.6 (standard error: 4.8, 95 % confidence interval 42.2–61.1) days in TH group, and estimated mean survival day is 56.9 (standard error: 3.3, 95 % confidence interval 50.3–63.4) days in the CAG group, respectively. As shown in the two figures, there is no significant difference in 90-day survival between the two groups although it tended to be better in the CAG group than in the TH group.

Table 3 shows the 90-day neurological prognosis of patients. As shown in Table 3, there is no significant difference in the rate of patients with good neurological prognosis at 90 days between the two groups.

**Table 3:** Primary outcome: 90-day neurological situation

	With and without coronary artery treatment			With coronary artery treatment		
	TH group (*N* = 76)	CAG group (*N* = 145)	*p*	TH group (*N* = 52)	CAG group (*N* = 112)	*p*
CPC1+2 [N (%)]	20 (26.3)	45 (31.0)	0.655	14 (26.9)	26 (23.2)	0.709

### Secondary outcomes

The secondary outcomes are shown in Table 4.

**Table 4:** Secondary outcome

	With and without coronary artery treatment			With coronary artery treatment		
	TH group (*N* = 76)	CAG group (*N* = 145)	*p*	TH group (*N* = 52)	CAG group (*N* = 112)	*p*
30-day neurological status CPC1+2 [*N* (%)]	23 (30.3)	50 (34.5 %)	0.672	17 (32.7)	32 (23.6)	0.729
With complications of hypothermia [*N* (%)]	24 (31.6)	41 (28.3)	0.768	22 (42.3)	29 (25.9)	0.177
Death caused by complications	3 (3.9)	5 (3.4)	1	2 (3.8 %)	3 (2.7 %)	0.655

Regardless of the coronary treatment, patients whose CPC are 1 or 2 are 23 (30.3 %) patients in the TH group, and 50 (34.5 %) patients in the CAG group in 30 days, respectively. Complications of hypothermia are seen in 24 (31.6 %) patients in the TH group, and 41 (28.3 %) patients in the CAG group between 90 days, and 3 (3.9 %) died from complications of hypothermia in the TH group, and 5 (3.4 %) were died in the CAG group.

Patients with coronary treatment, patients whose CPC were 1 or 2 are 17 (32.7 %) patients in the TH group, and 32 (28.6 %) patients in the CAG group in 30 days, respectively. The complications of hypothermia are seen in 22 (42.3 %) patients in the TH group, and in 29 (25.9 %) patients in the CAG group between 90 days. Two (3.8 %) died from complications of hypothermia in the TH group, and 3 (2.7 %) died in the CAG group.

There are no significant differences in the rate of good neurological prognosis at 30 days, complications of hypothermia, and death caused by complications of hypothermia between the two groups.

## Discussion

All healthcare workers involved in emergency medicine have been trying to improve the prognosis of patients with cardiac arrest. Recently, it had been reported that hypothermia was an effective treatment to improve the neurological situation and survival of comatose patients after ROSC [1, 2], and the usefulness of hypothermia recommended in the 2005 AHA Guidelines for CPR and ECC. Thereafter, the effectiveness of TH for comatose patients after ROSC has often been reported [15–17], and the 2010 AHA Guidelines for CPR and ECC recommended performing TH in comatose adult patients with ROSC after OHCA (class I–IIb) [3].

On the other hand, Spaulding et al. [4] report that accurate diagnosis by immediate CAG will yield suitable candidates for coronary angioplasty, and it seems to improve survival in patients with OHCA. Thereafter, the number of papers that reported the effectiveness of early CAG with potential coronary artery treatment in patients with ROSC after OHCA increased [4, 18–20], and the usefulness of early CAG and coronary artery treatment was described in the 2010 AHA Guidelines for CPR and ECC [3]. Recent reports have recommended both TH and CAG with coronary artery treatment [17, 21].

It seems to be ideal to start both TH and CAG in patients with OHCA immediately after arrival. However, there are doctors who concerned that there is an increase in pulmonary edema with the use of low-temperature infusion [7]. It is difficult for such doctors to start both TH and CAG at the same time. Additionally, all hospitals cannot always conduct immediate CAG for patients after ROSC. However, TH could be started if physicians experienced with TH after ROSC happen to be in the hospital. On the other hand, for CAG, cardiologists who are familiar with CAG are necessary. Many emergency physicians, cardiologists and intensivists have struggled with the clinical question of whether to start TH or CAG first after ROSC; however, there is no description about that in the 2010 AHA Guidelines for CPR and ECC. Therefore, we evaluated this point using data of the SOS-KANTO 2012 study.

We find that there are no statistically significant differences in survival and the 90-day neurological prognosis as primary outcomes, and in the 30-day neurological prognosis as the secondary outcome between the two groups regardless of coronary artery treatment. However, our finding that the survival rate tended to be better in the CAG group might have been influenced by type-II error because there were a small number of patients that were finally targeted for analysis. In addition, this result might indicate the importance of stabilizing hemodynamics first, so the survival curve of the CAG group may be better than that of the TH group without a statistical significant difference. On the other hand, Laver et al. [22] report that two-thirds of patients who die after OHCA die due to neurological injury and mortality is not associated with the ECG wave form of the cardiac arrest. In the present study, there is no significant difference in the 90-day neurological prognosis, and this result may have influenced the survival rate.

Regarding the secondary outcome, there are no significant differences in the rate of complications associated with hypothermia. This indicates that CAG can be performed safely with TH.

Temperature management for patients after ROSC is important. In this study we could not discern what temperature of hypothermia is best. The report of TTM trial investigators mentions that there are no significant differences between 33 and 36 °C in patients who are treated with temperature management after ROSC [23]. In their report, the Kaplan–Meier curves of both groups are almost same. On the other hand, Kaplan–Meier curve of the CAG group is better than the TH group (of course there is no significant difference as pointed out above), so there is a possibility that CAG first produces a better outcome than TH first in OHCA patients.

## Limitations

The SOS-KANTO study 2012 was a large, prospective cohort study of OHCA; however, the number of patients who underwent both TH and CAG was small, and this was only an observational study. This study did not include all hospitals with an emergency center in the KANTO area in Japan, and there is a possibility that our results are influenced by type-II error. There may have been differences in the indication to perform CAG and coronary artery treatment among the hospitals. In addition, the methods and apparatus for TH were varied and not unified. Therefore, a randomized double-blind trial would be preferable.

## Conclusion

Whether TH or CAG was performed earlier did not affect the survival and neurological prognosis after 90 days and 30-day neurological prognosis among patients with ROSC after OHCA. Regarding the priority of TH or CAG, either TH or CAG can be immediately started in the hospital for comatose patients with ROSC after OHCA.

## Appendix

The SOS-KANTO 2012 study group members: Shuichi Hagiwara, Kiyohiro Oshima, Kei Hayashida, Atushi Sakurai, Yoshio Tahara, Ken Nagao, Naohiro Yonemoto, Arino Yaguchi, and Naoto Morimura.

## References

[1] Hypothermia after cardiac arrest study group (2002). Mild therapeutic hypothermia to improve the neurologic outcome after cardiac arrest. N Engl J Med.

[2] Bernard SA, Gray TW, Buist MD, Jones BM, Silvester W, Gutteridge G, Smith K (2002). Treatment of comatose survivors of out-of-hospital cardiac arrest with induced hypothermia. N Engl J Med.

[3] Peberdy MA, Callaway CW, Neumar RW, Geocadin RG, Zimmerman JL, Donnino M, Gabrielli A, Silvers SM, Zaritsky AL, Merchant R, Vanden Hoek TL, Kronick SL (2010). Part 9: post-cardiac arrest care:2010 American heart association guidelines for cardiopulmonary resuscitation and emergency cardiovascular care. Circulation.

[4] Spaulding CM, Joly LM, Rosenberg A, Monchi M, Weber SN, Dhainaut JF, Carli P (1997). Immediate coronary angiography in survivors of out-of-hospital cardiac arrest. N Engl J Med.

[5] Reynolds JC, Callaway CW, El Khoudary SR, Moore CG, Alvarez RJ, Rittenberger JC (2009). Coronary angiography predicts improved outcome following cardiac arrest: propensity-adjusted analysis. J Intensive Care Med.

[6] Sunde K, Pytte M, Jacobsen D, Mangschau A, Jensen LP, Smedsrud C, Draegni T, Steen PA (2007). Implementation of a standardized treatment protocol for post resuscitation care after out-of-hospital cardiac arrest. Resuscitation.

[7] Kim F, Nichol G, Maynard C, Hallstrom A, Kudenchuk PJ, Rea T, Copass MK, Carlbom D, Deem S, Longstreth WT, Olsufka M, Cobb LA (2014). Effect of prehospital induction of mild hypothermia on survival and neurological status among adults with cardiac arrest: a randomized clinical trial. JAMA.

[8] SOS-KANTO study group (2015). Changes in pre- and in-hospital management and outcomes for out-of-hospital cardiac arrest between 2002 and 2012 in Kanto, Japan: the SOS-KANTO 2012 Study. Acute Med Surg.

[9] SOS-KANTO study group (2015). Changes in treatments and outcomes among elderly patients with out-of-hospital cardiac arrest between 2002 and 2012: a post hoc analysis of the SOS-KANTO 2002 and 2012. Resuscitation.

[10] Kitamura N, Nakada TA, Shinozaki K, Tahara Y, Sakurai A, Yonemoto N, Nagao K, Yaguchi A, Morimura N (2015). Subsequent shock deliveries are associated with increased favorable neurological outcomes in cardiac arrest patients who had initially non-shockable rhythms. Crit Care.

[11] Amino M, Inokuchi S, Nagao K, Nakagawa Y, Yoshioka K, Ikari Y, Funakoshi H, Hayakawa K, Matsuzaki M, Sakurai A, Tahara Y, Yonemoto N, Yaguchi A, Morimura N (2015) Nifekalant hydrochloride and amiodarone hydrochloride result in similar improvements for 24-hour survival in cardiopulmonary arrest patients: the SOS-KANTO 2012 study. J Cardiovasc Pharmacol 66:600–609. doi:10.1097/FJC.000000000000310 10.1097/FJC.0000000000000310 26317166

[12] Cummins RO, Chamberlain DA, Abramson NS, Allen M, Baskett PJ, Becker L, Bossaert L, Delooz HH, Dick WF, Eisenberg MS, Evans TR, Holmberg S, Kerber R, Mullie A, Ornat JP, Sandoe E, Skulberg A, Tunstall-Padoe H, Swanson R, Thies WH (1991). Recommended guidelines for uniform reporting of data from out-of-hospital cardiac arrest: the Utstein Style. A statement for health professionals from a task force of the American Heart Association, the European Resuscitation Council, the Heart and Stroke Foundation of Canada, and the Australian Resuscitation Council. Circulation.

[13] Jacobs I, Nadkarni V, Bahr J, Berg RA, Billi JE, Bossaert L, Cassan P, Coovadia A, D’Este K, Finn J, Halperin H, Handley A, Herlitz J, Hickey R, Idris A, Kloeck W, Larkin GL, Mancini ME, Mason P, Mears G, Monsieurs K, Montgomery W, Morley P, Nichol G, Nolan J, Okada K, Perlman J, Shuster M, Steen PA, Sterz F, Tibballs J, Timerman S, Truitt T, Zideman D (2004). Cardiac arrest and cardiopulmonary resuscitation outcome reports: update and simplification of the Utstein templates for resuscitation registries: a statement for healthcare professionals from a task force of the International Liaison Committee on Resuscitation (American Heart Association, European Resuscitation Council, Australian Resuscitation Council, New Zealand Resuscitation Council, Heart and Stroke Foundation of Canada, Inter American Heart Foundation, Resuscitation Councils of Southern Africa). Circulation.

[14] Kanda Y (2013). Investigation of the freely-available easy-to-use software “EZR” (Easy R) for medical statistics. Bone Marrow Transplant.

[15] Kern KB (2012). Optimal treatment of patients surviving out-of-hospital cardiac arrest. JACC Cardiovasc Interv.

[16] Zimmermann S, Flachskampf FA, Schneider R, Dechant K, Alff A, Klinghammer L, Rittger H, Achenbach S (2013). Mild therapeutic hypothermia after out-of-hospital cardiac arrest complicating ST-elevation myocardial infarction: long-term result in clinical practice. Clin Cardiol.

[17] Stub D, Hengel C, Chan W, Jackson D, Sanders K, Dart AM, Hilton A, Pellegrino V, Shaw JA, Duffy SJ, Bernard S, Kaye DM (2011). Usefulness of cooling and coronary catheterization to improve survival in out-of hospital cardiac arrest. Am J Cardiol.

[18] Cronier P, Vignon P, Bouferrache K, Aegerter P, Charron C, Templier F, Castro S, El Mahmoud R, Lory C, Pichon N, Dubourg O, Vieillard-Baron A (2011). Impact of routine percutaneous coronary intervention after out-of-hospital cardiac arrest due to ventricular fibrillation. Crit Care 2011.

[19] Callaway CW, Schmicker RH, Brown SP, Albrich JM, Andrusiek DL, Aufderheide TP, Christenson J, Daya MR, Falconer D, Husa RD, Idris AH, Ornato JP, Rac VE, Rea TD, Rittenberger JC, Sears G, Stiell IG (2014). Early coronary angiography and induced hypothermia are associated with survival and functional recovery after out-of-hospital cardiac arrest. Resuscitation.

[20] Vaillant C, Leurent G, Garlantezec R, Thebault C, Martins R, Bot E, Coudert I, Boulmier D, Le Breton H, Bedossa M (2013). Coronary angioplasty is associated with a better nerurological outcome in the era of modern management if out-of-hospital cardiac arrest. Int J Cardiol.

[21] Dumas F, White L, Stubbs BA, Cariou A, Rea TD (2012). Long-term prognosis following resuscitation from out of hospital cardiac arrest. J Am Coll Cardiol.

[22] Laver S, Farrow C, Turner D, Nolan J (2004). Mode of death after admission to an intensive care unit following cardiac arrest. Intensive Care Med.

[23] Nielsen N, Wetterslev J, Cronberg T, Erlinge D, Gasche Y, Hassager C, Horn J, Hovdenes J, Kjaergaard J, Kuiper M, Pellis T, Stammet P, Wanschcher M, Wise MP, Aneman A, Al-Subaie N, Boesgaard S, Bro-Jeppesen J, Brunetti I, Bugge JF, Hingston CD, Juffermans NP, Koopmans M, Kober L, Langorgen J, Lilja G, Moller JE, Rundgren M, Rylander C, Smid O, Werer C, Winkel P, Friberg H (2013). Target temperature management at 33 °C versus 36 °C after cardiac arrest. N Engl J Med.

